# Dendritic Scaffold onto Titanium Implants. A Versatile Strategy Increasing Biocompatibility

**DOI:** 10.3390/polym12040770

**Published:** 2020-04-01

**Authors:** Noemi Molina, Ana González, Donato Monopoli, Belinda Mentado, José Becerra, Leonor Santos-Ruiz, Yolanda Vida, Ezequiel Perez-Inestrosa

**Affiliations:** 1 Universidad de Málaga - IBIMA, Dpto. Química Orgánica, Campus de Teatinos s/n, 29071 Málaga, Spain; nmolina@uma.es; 2 Centro Andaluz de Nanomedicina y Biotecnología-BIONAND. Parque Tecnológico de Andalucía, c/ Severo Ochoa, 35, 29590 Campanillas, Málaga, Spainbecerra@uma.es (J.B.); 3 Universidad de Málaga - IBIMA, Dpto. Biología Celular, Genética y Fisiología, Facultad de Ciencias, Campus de Teatinos s/n, 29071 Málaga, Spain; 4 Departamento de Ingeniería Biomédica. Instituto Tecnológico de Canarias. c/ Añeta esq. Tigotán, 35118 Agüimes, Las Palmas, Spain; dmonopoli@itccanarias.org (D.M.); bmentado@itccanarias.org (B.M.); 5 Osteobionix s.l. c/ Ramón y Cajal 23, 35001 Las Palmas de Gran Canaria, Spain; 6 Centro de Investigación Biomédica en Red - Bioingeniería, Biomateriales y Nanomedicina (CIBER-BBN), 28029 Madrid, Spain

**Keywords:** dendritic structures, titanium implants, tripeptide arginine-glycine-aspartic acid (RGD) recognition pattern

## Abstract

Osseointegration of metal prosthetic implants is a yet unresolved clinical need that depends on the interplay between the implant surface and bone cells. The lack of a relationship between bone cells and metal has traditionally been solved by coating the former with “organic” ceramics, such as hydroxyapatite. A novel approach is hereby presented, immobilizing covalently dendrimeric structures onto titanium implants. Amide-based amino terminal dendrons were synthetized and coupled to titanium surfaces in a versatile and controlled way. The dendritic moieties provide an excellent scaffold for the covalent immobilization of bioactive molecules, such as extracellular matrix (ECM) protein components or antibiotics. Herein, tripeptide arginine-glycine-aspartic acid (RGD) motifs were used to decorate the dendritic scaffolds and their influence on cell adhesion and proliferation processes was evaluated.

## 1. Introduction

Titanium alloys are widely used in Orthopedics and Maxillofacial surgery to treat bone defects. However, the long-term integration of metallic prosthetic implants is a yet unresolved issue due to both biomechanical bone-metal mismatch, and lack of osseointegration, i.e., lack of chemical and structural bonding between the prosthesis and the bone where it is anchored. Another potential problem is that unmodified Ti is susceptible to bacterial infections, which could lead to complications or even to implant failure [1].

The advent of metal 3D printing by laser or electron beam melting has led to the possibility of better control over the biomechanical properties of prosthetic implants, as these technologies allow the tuning of the geometry and porosity of the prostheses. Osseointegration, on the other hand, depends on the interfacial relationship between the bone and the surface of the prosthesis. Cells do not recognize metal as an organic substrate and, consequently, permanent bonding between bone and implant does rarely occur. Surface modification has been proposed to overcome this restraint, and some prostheses already in the market present hydroxyapatite coatings to offer bone cells with a more recognizable substrate with which cells can interact [2,3].

Titanium is among the most studied and used materials in prosthetics due to its good biocompatibility, strong mechanical properties, high immunity to corrosion and complete inertness to the body environment [4]. The global dental implants and prosthetics market is worth billions of dollars each year. Musculoskeletal disorders are among the most widespread human health problems and now that we are facing a globally aging population, there has been an increase in orthopedic research. As a result, great efforts are being made in the development of new titanium implants that will improve the lifespan and lessen rejection rates of biomaterials.

In this regard, research focuses on improvement of the biocompatibility of the titanium implants to avoid not only an inflammatory response that would promote the rejection of the implant, but also the formation of fibrous tissue on the material surface that would hinder osseointegration and also promote bacterial infections [5]. Chemistry and topography play a crucial role in this sense. Modification of titanium surfaces has been traditionally carried out employing mechanical, physical and chemical treatments such as polishing, blasting, etching, oxidation and titanium plasma spraying [1,6,7]. However, the most studied strategy to favor osseointegration and diminish the risk of adverse tissue response is the immobilization of bioactive molecules on the titanium surfaces. Thus, growth factors, peptides, cell adhesive proteins, or polysaccharides have been immobilized over Ti surfaces to promote or enhance cell attachment [8]. Different methods have been used for the coating of biomolecules onto surfaces, such as polymer encapsulation, entrapment, physical adsorption and covalent or ionic binding. Nevertheless, covalent attachment presents the advantage of stable immobilization. The strong bond to the surface makes the biomolecules difficult to remove by wear and tear and robust enough to resist in vivo exposure [9].

Extracellular matrix (ECM) protein components have been extensively studied as promoters of specific binding interactions between the implant material and host cells. Among these, the tripeptide arginine-glycine-aspartic acid (RGD), a cellular recognition motif present in fibronectin and other ECM molecules, is among the most widely used [10,11]. Common techniques for the immobilization of RGD on implant surfaces involve the creation of self-assembled monolayers or polymer coating [12]. While different polymers have been tested, increasing interest has focused on dendrimers, a unique category of polymeric material. The 3D architecture of dendrimeric systems confers them intrinsic features including structural homogeneity, integrity, controlled composition and high-density multidentate homogeneous terminal groups available for bioconjugation. These unique properties make dendrimeric systems attractive for a variety of bioapplications [13,14] and have resulted in the growth of dendrimers as therapeutic tools in regenerative medicine in recent years [15]. The advantage over linear polymer is their regular branching structure, which provides an excellent scaffold for the incorporation of bioactive molecules such as ECM protein components, resulting in excellent recognition platforms. In our previous studies, we demonstrated that the modification of surfaces with RGD-decorated dendrimers promoted and enhanced cell adhesion [16]. Moreover, studies of RGD-decorated dendrimer nanopatterning showed that cell adhesion and proliferation are affected by local RGD surface density, differing from the observed results when using a homogenous linear layer of RGD peptides. The local RGD surface density acts as a regulator of chondrogenic commitment, and intermediate adhesiveness of cells to the substrates favors mesenchymal cell condensation and early chondrogenic differentiation [17,18].

Based on these results, we report the covalent binding of RDG-dendritic structures to titanium surfaces. We hypothesized that surface modification with these 3D recognition platforms would increase cell adhesion and proliferation processes, improving the biocompatibility of the titanium implants. Here, we present a bioactive surface based on the covalent immobilization of amide-based dendrons on Ti substrates. The amino terminal dendritic structures provide an excellent scaffold for the covalent immobilization of bioactive molecules, such as ECM protein components and antibiotics. Herein, the RGD motifs have been used to decorate the dendritic scaffolds and the influence of cell adhesion and proliferation processes was evaluated. The presented methodology resulted in a versatile and reproducible method for the covalent immobilization of bioactive molecules on Ti implants.

## 2. Materials and Methods

### 2.1. D-Printing of Titanium Disks

Disks of titanium alloy, measuring ø = 9 mm × 1 mm height, and regularly perforated by ø = 0, 9 mm pores, were manufactured by electron beam melting (EBM*^®^*) additive manufacturing. Disks were designed with Creo Parametric 3D modeling software (6.0, PTC; Boston, MA, USA) and manufactured from Ti_6_Al_4_V extralow interstitials (ELI) powder with 45–70 µm granulometry. Additive manufacturing was performed by successive fusion of slices that are 70 µm in thickness by means of a high-power electron beam operating in vacuum (pressure ≈1 × 10^−5^ mbar). After fusion, unmelted powder was cleaned away from the built disks by vacuum cleaning and sandblasting. A further cleaning step included ultrasound bathing in bi-distilled water. Titanium powder and all the machines used in the sintering procedure were purchased from Arcam (Mölnlycke, Göteborg, Sweden).

### 2.2. General Materials and Methods

All reactions were performed using commercially available reagents and solvents from the manufacturer without further purification. Unless otherwise stated, all reactions were performed in air. The titanium surfaces were oxidized using a mixture of sulfuric acid and hydrogen peroxide [19]. The synthesis of 3-maleimidopropionic acid N-hydroxysuccinimide ester and the preparation of phosphate buffered saline (PBS) are described elsewhere [20,21]. Column chromatography and TLC were performed on silica gel 60 (0.040–0.063 mm) using ultraviolet (UV) light and/or stains to visualize the products. Nuclear magnetic resonance (NMR) spectra were measured in the indicated deuterated solvent at 25 °C on a Bruker Ascend 400 MHz spectrometer. Proton chemical shifts (δ) are reported with the solvent resonance employed as the internal standard (CDCl_3_ δ 7.26, DMSO-*d_6_* δ 2.50, D_2_O δ 4.79, MeOD-*d*_4_ δ 3.31). Data are reported as follows: chemical shift, multiplicity, coupling constants (Hz) and integration. Carbon chemical shifts are reported in ppm with the solvent resonance as the internal standard (CDCl_3_ δ 77.16, DMSO-*d*_6_ δ 39.52, MeOD-*d*_4_ δ 49.00). The high-resolution mass spectrometry (HRMS) and electrospray ionization time of flight (ESI-TOF) mass spectra (MS) were performed on a High-Resolution Mass Spectrometer Orbitrap, Q-Exactive (Thermo Fisher Scientific, Waltham, MA, USA), in either the positive or negative ion mode. Hydrogenolysis reactions were carried out under hydrogen atmosphere (50 bar) using a Mini-Reactor from Erie-Autoclave Engineers (Erie, PA, USA). X-ray photoelectron spectroscopy (XPS) data were obtained from Multilab System 2000 (Thermo Fisher). UV–visible spectra were performed on a Cary 100 Bio UV–Visible Spectrophotometer (Sata Clara, CA, USA). All data were represented as the mean values ± standard deviations. Statistical analyses were performed using the Mann–Whitney Rank Sum Test and Student’s *t*-test quadruplicate. Statistical significance was considered for *p*-values ≤ 0.05. Data were analyzed with SigmaPlot 12.5 software (2013, Systat Software, Chicago, IL, USA).

### 2.3. Synthesis of 3,3′-Diazidopivalic Acid *(**2**)*

Sodium azide (7.50 g, 115 mmol) was added to a solution of 3,3′-dichloropivalic acid (5.00 g, 29 mmol) in *N,N*-Dimethylformamide (DMF) /H_2_O 9:1 (20 mL). The resulting solution was heated in a heat block at 80 °C overnight. The solvent was removed under vacuum and ethyl acetate (50 mL) was added to promote the precipitation of the remaining sodium azide. The mixture was filtered, and the solvent was removed under vacuum to obtain the product (4.80 g, 90%) as a colorless oil. ^1^H NMR (400 MHz, CDCl_3_, δ): 3.63 (d, *J* = 12.3 Hz, 2 H, CH_2_), 3.52 (d, *J* = 12.3 Hz, 2 H, CH_2_), 1.27 (s, 3 H, CH_3_). ^13^C NMR (100 MHz, CDCl_3_, δ): 179.8, 54.6, 47.6, 19.4. HRMS (ESI) *m*/*z*: (M − H)^–^ calcd for C_5_H_7_N_6_O_2_^–^, 183.0625; found, 183.0626.

### 2.4. Synthesis of Benzyl-3,3′-Diazidopivaloate *(**3**)*

To a solution of 2 (5.0 g, 27 mmol) in DMF (20 mL), sodium carbonate (4.29 g, 40.5 mmol) and benzyl bromide (4.8 mL, 40.5 mmol) were added. The mixture was stirred overnight at room temperature. Then, hexane (100 mL) and water (50 mL) were added and the phases were separated. The organic phase was washed with water (3 × 50 mL) and dried over MgSO_4_ and then the solvent was removed under vacuum. Purification was performed by silica gel column chromatography (hexane/ethyl acetate, 9:1 *v*/*v*) to obtain the product (6.66 g, 90%) as a colorless oil. ^1^H NMR (400 MHz, CDCl_3,_ δ): 7.42–7.32 (m, 5 H, Ar H), 5.19 (s, 2 H, O–CH_2_), 3.63 (d, *J* = 12.2 Hz, 2 H, N_3_–CH_2_), 3.52 (d, *J* = 12.2 Hz, 2 H, N_3_–CH_2_) 1.24 (s, 3 H, CH_3_). ^13^C NMR (100 MHz, CDCl_3_, δ): 173.1, 135.4, 128.8, 128.6, 128.3, 67.3, 54.9, 47.8, 19.4. HRMS (ESI) *m*/*z*: (M + Na)^+^ calcd for C_12_H_14_N_6_O_2_Na^+^, 297.1070; found, 297.1072.

### 2.5. Synthesis of Benzyl-3,3’-Diaminopivaloate *(**4**)*

Compound **3** (500 mg, 1.82 mmol) was dissolved in tetrahydrofuran (THF) (10 mL) and placed in an ice bath. Triphenylphosphine (2.50 g, 9.65 mmol) was dissolved in THF (5 mL) and added dropwise to the solution of 3. The mixture was left under reflux in a heat block overnight. Afterwards, 0.3 mL of water was added and the reaction was left under reflux for another day. Then, THF was removed under vacuum and the product was dissolved in 1 M HCl (6 mL). The aqueous phase was washed with dichloromethane (5 × 20 mL). The water was later removed under vacuum to obtain 4 (527 mg, 98%) as a colorless solid. ^1^H NMR (400 MHz, D_2_O, δ): 7.56–7.43 (m, 5 H, CH Ar), 5.35 (s, 2 H, O–CH_2_), 3.45 (d, *J* = 13.7 Hz, 2 H, NH_2_–CH_2_), 3.29 (d, *J* = 13.7 Hz, 2 H, NH_2_–CH_2_), 1.49 (s, 3 H, CH_3_). ^13^C NMR (100 MHz, MeOD-*d*_4_, δ): 173.2, 136.3, 129.63, 129.61, 129.59, 69.4, 45.0, 44.34, 19.4. HRMS (ESI) *m*/*z*: [M + H]^+^ calcd for C_12_H_19_N_2_O_2_^+^, 223.1441; found, 223.1441.

### 2.6. Synthesis of Benzyl-3,3´-Bis(Tert-Butoxycarbonyl) Aminopivaloate *(**5**)*

To an ice-cooled solution of **4** (200 mg, 0.90 mmol) in H_2_O/acetone at a 1:1 ratio (10 mL), 1 M NaOH was added dropwise until pH > 10 was achieved. Di-*tert*-butyl dicarbonate (393 mg, 1.80 mmol) was then added and the reaction was stirred overnight at room temperature. The product was extracted using dichloromethane (4 × 25 mL). The organic phase was dried with MgSO_4_ and the solvent was removed under vacuum to obtain the product (365 mg, 96%) as a colorless solid. ^1^H NMR (400 MHz, CDCl_3_, δ): 7.39–7.30 (m, 5 H, Ar CH), 5.14 (s, 2 H, O–CH_2_), 3.48 (dd, *J* = 14.4, 8.6 Hz, 2 H, NHBoc–CH_2_), 3.12 (dd, *J* = 14.4, 5.2 Hz, 2 H, NHBoc–CH_2_), 1.43 (s, 18 H, Boc CH_3_), 1.14 (s, 3 H, CH_3_). ^13^C NMR (100 MHz, CDCl_3_, δ): 175.2, 156.7, 135.8, 128.7, 128.4, 128.0, 79.4, 66.7, 48.9, 43.5, 28.4, 19.0. HRMS (ESI) *m*/*z*: (M + Na)^+^ calcd for C_22_H_34_N_2_O_6_Na^+^, 445.2309; found, 445.2309.

### 2.7. Synthesis of 3,3´-Bis(Tert-Butoxycarbonyl) Aminopivalic Acid *(**6**)*

To a solution of **5** (400 mg, 0.95 mmol) in methanol (10 mL), Pearlman’s catalyst (100 mg, 0.71 mmol) was added. After hydrogenation for two hours, the catalyst was removed by filtration through MeOH-pre-wetted celite. The solvent was removed under vacuum to obtain **6** (309 mg, 98%) as a colorless solid. ^1^H NMR (400 MHz, MeOD-*d*_4_, δ): 3.25 (d, *J* = 14.2 Hz, 2 H, CH_2_), 3.17 (d, *J* = 14.2 Hz, 2 H, CH_2_), 1.43 (s, 18 H, Boc CH_3_) 1.07 (s, 3 H, CH_3_). ^13^C NMR (100 MHz, DMSO-*d*_6_, δ): 180.0, 158.7, 80.2, 46.7, 45.5, 28.7, 19.6. HRMS (ESI) *m*/*z*: (M + Na)^+^ calcd for C_15_H_28_N_2_O_6_Na^+^, 355.1840; found, 355.1838.

### 2.8. Synthesis of Compound ***7***

A solution of **6** (644 mg, 1.94 mmol) in anhydrous acetonitrile (3 mL) was added to a solution of 1,1-carbonyldiimidazole (CDI) (315 mg, 1.94 mmol) in anhydrous acetonitrile (3 mL) and the mixture was stirred at room temperature for one hour. Afterwards, a solution of 4 (215 mg, 0.96 mmol) was added and the stirring mixture was left overnight at room temperature. The solvent was removed under vacuum and the residue was dissolved in dichloromethane (40 mL) and washed with 0.05 M HCl (3 × 30 mL). The combined organic phase was dried with MgSO_4_, filtered and concentrated under reduced pressure to obtain the product (678 mg, 83%) as a colorless solid. ^1^H NMR (400 MHz, CDCl_3_, δ): 7.40–7.29 (m, 5 H, Ar CH), 5.14 (s, 2 H, O–CH_2_), 3.51–3.06 (m, 12 H, CH_2_), 1.42 (s, 36 H, Boc CH_3_), 1.17–105 (m, 9 H, CH_3_). ^13^C NMR (100 MHz, CDCl_3_, δ): 174.9, 157.2, 130.1, 128.8, 128.6, 128.5, 79.6, 67.3, 48.6, 48.0, 44.8, 42.2, 28.5, 19.4. HRMS (ESI) *m*/*z*: (M + Na)^+^ calcd for C_42_H_70_N_6_O_12_ Na^+^, 873.4944; found, 873.4944.

### 2.9. Synthesis of Compound ***1***

To a solution of **7** (515 mg, 0.61 mmol) in methanol (10 mL), Pearlman’s catalyst (60 mg, 0.44 mmol) was added. After hydrogenation for two hours, the catalyst was removed by filtration through MeOH-pre-wetted celite. The solvent was removed under vacuum to obtain **7** (460 mg, 98%) as a colorless solid. ^1^H NMR (400 MHz, MeOD-*d*_4_, δ): 3.48–3.20 (m, 12 H, CH_2_), 1.44 (s, 36 H, Boc CH_3_) 1.18–1.03 (m, 9 H, CH_3_). ^13^C NMR (100 MHz, MeOD-*d*_4_, δ): 177.5, 158.7, 80.3, 49.8, 46.0, 45.3, 43.7, 28.8, 20.3, 19.5. HRMS (ESI) *m*/*z*: (M + Na)^+^ calcd for C_35_H_64_N_6_O_12_Na^+^, 783.4474; found, 783.4481.

### 2.10. Preparation of Ti disk (VII)

#### 2.10.1. Preparation of Ti Disk (I)

The disks were immersed for 2 h in a solution of concentrated H_2_SO_4_ and 30% aqueous H_2_O_2_ at a 1:1 *v*/*v* ratio (100 mL). Afterwards, they were rinsed with distilled water.

#### 2.10.2. Preparation of Ti Disk (II)

The disks were immersed in a solution of (3-aminopropyl)trimethoxysilane in ethanol 1:3 *v*/*v* (100 mL) and were placed in an orbital agitator for four hours. Afterwards, the disks were washed with ethanol.

#### 2.10.3. Preparation of Ti Disk (III)

A solution of 1 (30 mg, 0.04 mmol, 1 eq) in anhydrous acetonitrile (2 mL) was added to a solution of 1,1′-carbonyldiimidazole (CDI) (7 mg, 0.04 mmol, 1 eq) in anhydrous acetonitrile (2 mL) and the mixture was stirred at room temperature for one hour. The disks were immersed in anhydrous acetonitrile and the mixture was added. They were placed in an orbital agitator overnight. Afterwards, they were washed with ethanol.

#### 2.10.4. Preparation of Ti Disk (IV)

The disks were immersed in a solution of glycidol (5 µL, 6.5 × 10^−2^ mmol, 1000 eq) in 2-propanol (60 mL) and placed in an orbital agitator overnight. Afterwards the disks were washed with 2-propanol and dichloromethane.

#### 2.10.5. Preparation of Ti Disk (V)

The reaction was carried out under nitrogen atmosphere. The disks were immersed in a solution of 4 M hydrochloric acid in dioxane/THF at 1:1 ratio (20 mL) and were placed in an orbital agitator for two hours. Afterwards, they were washed with THF and immersed in a solution of sodium hydroxide 10% for one hour. The disks were washed with acetone.

#### 2.10.6. Preparation of Ti Disk (VI)

The disks were immersed in an ice-cooled solution of *N,N*-Diisopropylethylamine (DIPEA) (3 mL, 1000 eq) in DMF (60 mL). A solution of 3-maleimidopropionic acid *N*-hydroxysuccinimide ester (50 mg, 1000 eq) in DMF (3 mL) was added and the disks were placed in an orbital agitator overnight. The disks were washed with DMF and acetone.

#### 2.10.7. Preparation of Ti Disk (VII)

The reaction was carried out under argon atmosphere. A solution of RGD-Cys (2 mg, 4.4 eq) in PBS (10 mL) was added to the disks. They were placed in an orbital agitator for two days. Afterwards, the disks were washed with PBS, water and acetone.

### 2.11. Quantification of Free Primary Amino Groups Bonded to the Ti disk

The determination of the free primary amino groups present on the titanium disks was carried out by using a previously described ninhydrin procedure [22]. Measurements were carried out with half-disk in duplicate, resulting in reproducible values. The media of the absorbance values obtained are 0.05 for Ti disk (II); 0.006 for Ti disk (III); 0.004 for Ti disk (IV); 0.02 for Ti disk (V) and 0.0008 for Ti disk (VI).

### 2.12. Culture of Osteoblastic Cells on the Ti_6_Al_4_V ELI

Human osteoblastic cells were used to evaluate the effect of RGD dendrimer on cell adhesion and proliferation. Osteoblasts were purchased from Sigma-Aldrich (St. Louis, MO, USA). The cells were cultured in Alpha Minimum Essential Medium Eagle (alpha-MEM) with ribonucleosides, deoxyribonucleosides and sodium bicarbonate supplemented with 100 U/mL penicillin, 100 µg/mL streptomycin, 1.25 µg/mL amphotericin B, 2 mM L-glutamine, and 10% Fetal Bovine Serum (FBS). All the samples were incubated under 5% CO_2_ atmosphere at 37 °C and the medium was replaced every 2 days. The cells used for the essays were at passage 2. Prior to cell seeding, titanium disks were sterilized by immersion in 70% ethanol for 30 min, and later washed with sterile bi-distilled water. They were then immersed in alpha-MEM at 4 °C for 90 min. All reagents were purchased from Sigma-Aldrich (St. Louis, MO, USA).

### 2.13. Cell Adhesion and Proliferation Essays

Resazurin reduction essay was used to estimate cell number in the analyzed samples. Four RGD-coated titanium disks and four uncoated disks (controls) were used in each essay (n = 4). To evaluate cell adhesion, the disks were placed on the wells of TC-grade, 48-well multiplates and each was seeded with 50,000 cells in the normal (10% FBS) growth medium or the low-serum medium (2% FBS). After 2 h, disks were transferred to new wells and the cells attached to them were measured by resazurin essay. This consisted of a 4 h incubation in the growth medium supplemented with 20% resazurin solution (resazurin sodium salt aqueous solution 440 mM), in standard culture conditions (37 °C, 5% CO_2_). After the incubation, absorbance of the resazurin-supplemented media was measured at 570 nm and 600 nm in an *Eon* microplate spectrophotometer (Biotek, Winooski, VT, USA). The reduction percentage of resazurin was calculated according to the alamarBlue^®^ protocol and U.S. Patent No. 5,501,959 (ThermoFisher Scientific, Waltham, MA, USA). A set of wells containing known cell densities (from 1500 to 100,000 cells/well) was seeded along with the disks in order to establish a “calibration line” that allowed the conversion of resazurin reduction percentage into cell number.

Subsequently, resazurin solution was replaced by the growth medium to continue the culture experiment. Assessment of cell growth on the scaffolds was carried out by repeating the resazurin reduction essay on the cell-seeded scaffolds at different time points: 3, 7, 14 and 21 days after seeding. Experiments were repeated three times.

Unless otherwise stated, all reagents used were purchased from Sigma-Aldrich (St. Louis, MO, USA). Data were subject to statistical analysis with SPSS software (SigmaPlot 12.5 software (2013, Systat Software, Chicago, IL, USA)).

### 2.14. Environmental Scanning Electron Microscopy (ESEM)

Osteoblastic cells seeded on the disks were imaged with environmental scanning electron microscopy (ESEM) to assess their morphology. For this purpose, disks were seeded as already described (50,000 cells/disk in normal medium) and, on days 1 and 3 after seeding, they were fixed for 30 min by immersion in 4% paraformaldehyde solution. This was freshly prepared by dissolving paraformaldehyde powder in prewarmed (60 °C) phosphate buffer (PB, pH = 7.4), which was allowed to cool down to room temperature before use. After fixation, samples were thoroughly washed in PBS and imaged in a FEI Quanta 250 FEG scanning electron microscope (Hillsboro, OR, USA).

## 3. Results

### 3.1. Modification of Ti Disk Surfaces

**Scheme 1:**
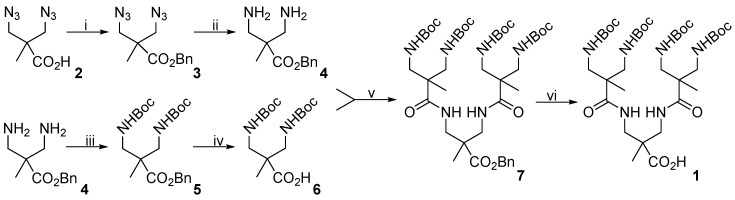
Synthesis of the generation **2** dendron (**1**): (i) BnBr, Na_2_CO_3_/ *N,N*-Dimethylformamide (DMF); (ii) PPh_3_/ tetrahydrofuran (THF),then H_2_O; (iii) Boc_2_O, NaOH/H_2_O:acetone; (iv) H_2_, Pd(OH)_2_ on carbon/MeOH; (v) 1,1-carbonyldiimidazole (CDI)/MeCN; (vi) H_2_, Pd(OH)_2_ on carbon/MeOH.

The functionalization of inorganic substrates by anchoring organic molecules to the surface is a very powerful method to control cell–material interactions and increase material biocompatibility [9]. To functionalize the titanium disk, we explored a novel approach involving covalent binding between the RDG scaffolds and the surfaces. A generation **2** dendron (**1**, Scheme 1) was designed in order to expose the linear R-G-D tripeptide to the media using multivalence, while its focal point was coupled covalently to the surface. Based on our previous experience in the synthesis of all-aliphatic polyamide dendrimers [23], a derivative structure was prepared using 3,3′-dichloropivalic acid as starting material (Scheme 1). The 3,3´-diazidopivalic acid was prepared under previously described procedures [23]. The synthetic procedure involved the protection of the carboxylic acid function as a benzylic ester and the reduction of the azido groups to yield the focal point of the dendron (Scheme 1). The 3,3´-bis(*tert*-butoxycarbonyl) aminopivalic acid (**6**, Scheme 1) was chosen as the branching unit. Coupling between both structures gives a generation **2** dendron as the result, with both the amino terminal groups and focal point protected. The deprotection of the carboxylic acid via a hydrogenation reaction yielded **1** (See Figures S1–S16 and Tables S1 and S2 in ESI).

**Figure 1:**
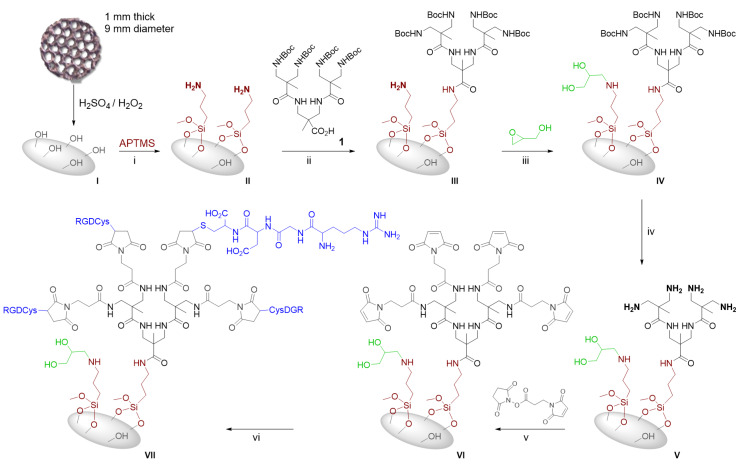
Covalent modification of Ti disk: (i) EtOH; (ii) CDI/CH_3_CN; (iii) propanol; (iv) HCl (4 M) in dioxane/THF; (v) *N,N*-Diisopropylethylamine (DIPEA) /DMF; (vi) phosphate buffer saline (PBS).

The complete the procedure for covalent binding between **1** and the titanium surface is illustrated in Figure 1. For this study, titanium disks that are 1 mm in thickness and 9 mm in diameter were used. Titanium surfaces are spontaneously covered by a titanium oxide layer of 3–6 nm thickness. However, this TiO_2_ coating is usually noncrystalline and irregular in thickness and composition [24,25]. In order to avoid reproducibility problems caused by this irregularity and increase the number of hydroxyl groups, titanium surfaces were oxidized with a H_2_SO_4_/H_2_O_2_ mixture. This controlled oxidation provides a clean surface homogeneously covered by hydroxyl groups, which are indispensable for chemical binding [19]. Silanization is undoubtedly the most widely used approach to introduce organic functional groups on inorganic surfaces [26]. In fact, silane based strategies have been previously used to couple organic compounds to titanium implants [27,28]. Here, we used silane modification in a slightly different way, with the aim of endorsing an exhaustive control on the derivatization process of the Ti surfaces with the dendrimeric moiety. The (3-aminopropyl) trimethoxysilane (APTMS) was used in order to introduce primary amino groups on the surface. These amino groups will be used for two objectives: as an anchoring point between the surface and the dendritic structure and as a powerful tool to control the degree of modification of the surface.

Titanium disks were first reacted with APTMS to obtain Ti disk (II) (Figure 1). The reaction of APTMS with the hydroxyl groups on the titanium surface was conducted in aqueous ethanol, since this methodology is reported to generate a thinner silane layer with a more even coverage of the surface [29]. To ensure the absence of noncovalent bound silane, disks were thoroughly washed with ethanol. The derivatization process was monitored via the quantification of the inserted primary amino groups with the ninhydrin test (Table 1) [22,30]. The second step in the functionalization process involved the reaction of the amino groups on the surface of Ti disk (II), with the carboxylic acid on the focal point of **1**. For amide formation, 1,1′-carbonyldiimidazole (CDI) was used as the coupling agent. To ensure covalent binding between the dendron and the surface, ninhydrin tests were used to evaluate changes in the amount of free amino groups present in Ti disk (III). The presence of amino groups decreases dramatically, which indicates a high degree of reaction. To ensure that no free amino groups remain in the disks and to increase the hydrophilicity of the Ti surface, a treatment with glycidol was performed to afford Ti disk (IV). Once the dendrons were covalently attached to the surface and residual free amino groups were blocked, the deprotection of the terminal groups of the dendrons was achieved. Ti disks (IV) were treated with hydrochloric acid in a dioxane/THF mixture to eliminate the tert-butoxycarbonyl protecting group. In order to neutralize the ammonium salt formed, a treatment with sodium hydroxide was carried out. The ninhydrin test confirms the huge increase in the number of free amino groups on the surface (Ti disk V, Table 1).

To decorate the peripheral dendron groups with peptide ligands, the use of efficient and chemoselective conjugation chemistry to ensure complete ligand attachment to the dendron is important. Based on our previous experience, cysteine residues bind maleimide-terminated dendrimers in an excellent and efficient manner [16]. In this regard, we used the terminal thiol group of the cysteine unit in the R-G-D-Cys tetrapeptide to introduce the R-G-D moiety in the disk. Dendrons were reacted with a β-alanine derivative to introduce the maleimido functionality. For this purpose, Ti disks (V) were treated with 3-maleimidopropionic acid *N*-hydroxysuccinimide ester. The reaction was monitored through the ninhydrin test, thus confirming the noticeable decrease in the number of primary amino groups, which becomes practically zero (Table 1).

The last step in the derivatization process consists of the Michael addition of the terminal thiol group of the Cys residue to the maleimido moieties. A solution of RDG-Cys in PBS was added to Ti disk (VI) and left under argon atmosphere for 48 h. After subsequent washes, Ti disks (VII) were obtained. To ensure the successful immobilization of the peptide on the titanium surface, the atomic composition of disk (VI) and (VII) was determined by XPS (Table 2). As expected, the XPS-determined elemental composition of the (VI) titanium surface showed the presence of titanium, oxygen, nitrogen and carbon components. After peptide functionalization (Ti disk (VII)), the presence of sulfur was also observed, which is consistent with the immobilization of the peptide on the surface.

**Figure 2:**
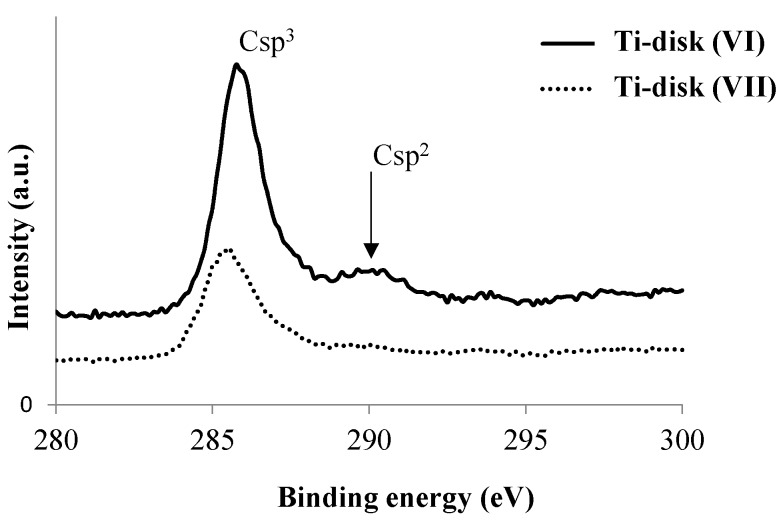
C1s X-ray photoelectron spectroscopy (XPS) spectra of Ti disk (VI) and Ti disk (VII).

Moreover, the C1s spectra of disks (VI) and (VII) show how the concentration of C sp^2^ decreases when RGD-Cys is coupled to the maleimide group present in the dendritic structure (Figure 2), which is consistent with the reaction between the maleimide and the thiol group.

### 3.2. Essays of RGD-Functionalized Titanium Surfaces with Pre-Osteoblastic Cells

**Figure 3:**
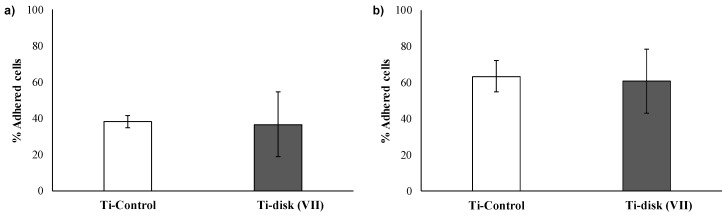
Adhesion of osteoblastic cells to nude Ti_6_Al_4_V extralow interstitials (ELI) disks (Ti Control) and tripeptide arginine-glycine-aspartic acid (RGD)-coated disks (Ti disk (VII)), in (**a**) the normal growth medium and (**b**) the low-serum medium.

As shown in Figure 3a, osteoblastic cells could adhere to both nude (control) and RGD-coated EBM-sintered Ti_6_Al_4_V ELI disks. Coating the metal surface with RGD-dendritic structures did not significantly improve the number of adhered cells. As serum proteins have been reported to interfere with osteoblastic adhesion [31,32,33], the essay was repeated in a low-serum medium. Reduction of serum concentration increased the percentage of adhered cells in both nude and RGD-coated titanium disks, but again no significant differences were found as to adhesion between RGD-coated and nude surfaces.

**Figure 4:**
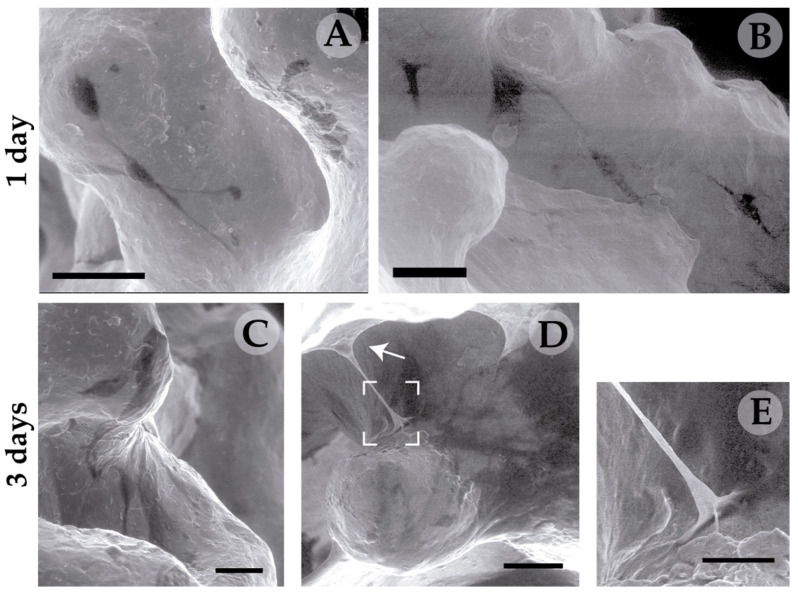
Environmental scanning electron microscopy (ESEM) of human osteoblastic cells seeded onto nude and RGD-coated titanium disks, 1 and 3 days after seeding. (**A**) and (**C**) correspond to Ti Control, while (**B**), (**D**) and (**E**) correspond to Ti disk (VII). The arrow in D points to a cell that is bridging the gap between two protrusions of the titanium surface, by means of a filopodium boxed in D and magnified in E. Bars: 50 µm in A-B, 25 µm in C-D, and 10 µm in E.

In accordance with the adhesion data, no differences were observed as to cellular morphology between nude and RGD-coated disks. As observed in Figure 4 (particularly 4a and 4b), osteoblastic cells displayed a fibroblastic shape, with elongated filopodia. This shape is characteristic of osteoblastic cells in pre-confluent cultures, where cells are spreading and multiplying. Three days after seeding (Figure 4c,d), cells were more abundant and they were adapted to the irregular profiles of the titanium surface, both in nude and RGD-coated titanium. Some cells have extended filopodia that could bridge the gap between the surface irregularities (arrow in Figure 4d).

**Figure 5:**
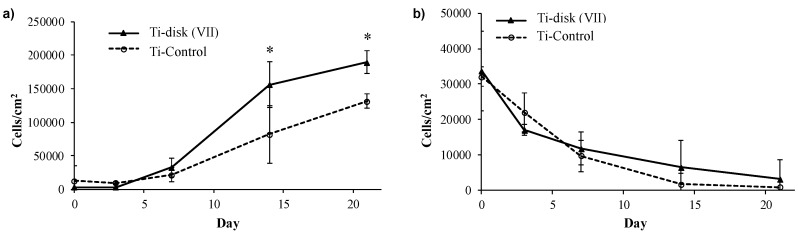
Proliferation of osteoblastic cells in nude Ti_6_Al_4_V ELI disks (Ti Control) and RGD-coated disks (Ti disk (VII)), in (**a**) the normal growth medium and (**b**) the low-serum medium. Asterisks indicate statistically significant differences (*p* < 0.005).

In contrast to adhesion, proliferation was found to be affected by the RGD coating of the disk surface. Figure 5 shows the growth curve of osteoblastic cells seeded on nude and coated titanium. In the normal growth medium, a significant increase in cell density was found in RGD-coated disks after seven days in culture, suggesting that RGD signaling is enhanced in these disks. Since integrin signaling can control both cell survival and proliferation, the trial was also performed in low-serum medium, which does not contain enough serum to allow osteoblastic survival in the mid and long term. As expected, cell growth was negative and the culture population decreased with time in both controls and RGD-coated disks, but a certain “rescue” effect could be attributed to RGD coatings, since cell density was greater in disks decorated with the RGD-dendritic structure, particularly at longer time points (7 and 14 days in culture).

## 4. Discussion

Dental implants have been in use for over five decades to treat edentualism and, despite a high degree of satisfactory results, a 6% failure rate still persists. Clinical failure is related to biomechanical overload, infection or a poor relationship between bone and implant, resulting in rejection, peri-implantitis, bone loss and implant loosening [34,35].

Our goal was contributing to improve the osseointegrative properties of dental implants, through a novel surface modification method that could facilitate the interfacial relationship between bone and implant. The premise was that the method should be tunable, offering the possibility of attaching different moieties according to specific needs, and should also be stable in order to resist the harsh conditions of the in vivo environment, particularly in the oral cavity. We opted for Ti_6_Al_4_V ELI alloy because it is widely used in the dental implant industry [36], and chose 3D-printed Ti_6_Al_4_V ELI pieces because metal 3D printing techniques are opening the field of implantology to increasingly improved designs in order to tackle the biomechanical issue [37,38,39].

The biocompatibility and safety of Ti_6_Al_4_V ELI and other titanium alloys are based on its corrosion resistance and virtually complete inertness to the body environment. However, biocompatibility based on inertness to biological ambients is a double-edged sword: on the one hand, there will be no adverse reactions based on inflammation of rejection but, on the other hand, there will be no recognition of the implant by the host living tissue and, as a result, no bonding between implant and bone, leading to defective osseointegration and proneness to infection. This issue has been approached from multiple perspectives, with most of them consisting on interposing a ceramic or glass coating between the metal surface and the living tissue [40]. More direct approaches have focused on modifying surface topography. Though multiple in vitro studies indicate that a certain degree of roughness favors cell attachment and differentiation, in vivo studies have shown variable results [40,41,42]. An alternative or complementary strategy is to decorate the implant surface with molecular moieties that offer cells with sites where they can interact. These would be ligands for membrane receptors, preferably those located in the extracellular matrix. In this context, the most used and studied motif is the RGD domain, a binding site for the integrin receptor, present in fibronectin and other extracellular matrix proteins [43,44].

Previous studies evaluating the influence of the RGD domain in cell adhesion and proliferation showed that the use of dendrimers as scaffolds to distribute the RGD domain through a surface promotes a higher interaction than a homogenous linear layer of RGD peptides [16]. Moreover, nanopatterns of RGD-decorated dendrimers showed that cell adhesion, proliferation and differentiation are affected by local RGD surface density [17,18]. Therefore, the way in which the RGD domain is exposed influences cell response. Although different dendrimer depositions onto titanium surfaces has been previously reported [45,46,47], in all cases, dendrimer coatings were carried out via physical adsorption. Despite the encouraging results obtained, physical adsorption is not enough to guarantee the stability and resistance needed in the case of dental implants. The exposure of the surface to oral conditions required the strong and stable immobilization of the biomolecule that provides the covalent attachment.

For this study, we selected a new type of dendritic structure as the scaffold that best suits covalent immobilization. A generation 2 amino terminal all-aliphatic amide-based dendron was selected as the most suitable scaffold, as its size and number of terminal amino groups available for peptide attachment should provide enough multivalence for effective cellular interaction. Additionally, the focal point allows covalent binding with the surface. The designed surface modification strategy has three fundamental advantages. First, the previous amino functionalization of the titanium surface allows coupling with the dendritic structure through the formation of an amide bond. This implies that all the organic dendritic structures that support the RGD domain (Figure 1) are based on amide bonds, a perfectly stable group under biological conditions. Thus, deposition of the dendritic scaffold is achieved in a strong, resistant and controlled manner. On the other hand, the derivatization procedure involves an increase or decrease in primary amino groups on the surface. This fluctuation allows monitorization of the process through the quantification of the primary amino groups present on the surface, which is a powerful tool to verify the correct functionalization of the titanium disk. Finally, once the dendron is covalently deposited (Ti disk V), the amino terminal groups give the possibility of conjugation with many different molecules. The derivatization procedure used allows not only a stable and versatile modification of titanium surfaces, but also enables assessing the quality of the material obtained. Reproducibility in surface composition and quality is a critical requirement for clinical applications.

In order to test whether dendritic-exposed biomotifs are indeed active, we undertook a proof-of-concept study, attaching the RGD peptide to the dendron bound to the surface of the titanium disks and then testing the behavior of osteoblastic cells on these disks. RGD is a common domain in multiple extracellular-matrix proteins and mediates the attachment of the cell to the substrate through integrin receptors. Signaling through integrin receptors not only mediates cell attachment, but also cell survival, proliferation and differentiation and, for this reason, it is commonly used in biomaterial surfaces [43,44]. Osteoblastic cells, characteristic of the bone tissue (where metal implants are anchored), were chosen to essay the biological effect of RGD-dendron binding to titanium alloy implants. We found that osteoblast adhesion was not modified by the presence of RGD motifs in the disk surface. This is not surprising, as osteoblast adhesion is mediated by several membrane receptors other than integrins, and even integrin-mediated attachment can occur through other domains, in addition to RGD [48,49,50].

Unlike cell adhesion, cell proliferation was positively affected by the presence of RGD motifs on the titanium surface. As mentioned earlier, integrin signaling is required for adherent cells to survive and it is also involved in cell proliferation. Contribution of integrins to cell multiplication occurs in multiple ways. Integrin signaling cooperates with growth factors to stimulate or sustain cell division, either by controlling the expression of growth factor receptors at the transcriptional level or by activating these receptors in the absence of its ligand. Integrins can also activate *Akt* signaling and the *Erk* pathway—both of which are mechanisms that drive G1 to S transition in adhered cells [51]. In osteoblastic cells, it was shown that integrin signaling activates AP-1 transcription factors *c-fos* and *c-jun*, thus stimulating the cell to enter the division cycle [52]. Establishing how integrin signaling is stimulating cell division in the case of RGD-dendron-decorated titanium is beyond the scope of this study, but our results prove that a significant increase in osteoblastic proliferation occurs in these disks, and therefore RGD signaling must be active.

This study opens up the possibility of using dendron attachment to decorate titanium implants with selected biological cues, which could be chosen according to the patient’s needs. These molecular signals could be intended to affect the behavior of resident bone cells, but also to prevent bacterial adhesion, as infection is a common cause of dental implant failure. This could also expose anti-inflammatory cytokines to prevent rejection or specific growth factors to stimulate bone formation around the implant.

## 5. Conclusions

A novel approach to increase the biocompatibility of titanium alloys was presented. Modification of titanium surface has been addressed through the covalent immobilization of dendrimeric structures. Amide-based amino terminal dendrons act as a perfect scaffold to present bioactive molecules through a surface that favors interaction with cells. The derivatization procedure has been designed in order to enable stable and versatile coating of titanium surfaces, which also enables assessing surface composition. Both the stability of the organic framework and the quality of the coating are crucial issues for applications in oral implants. Herein, RGD motifs were used to decorate the dendritic scaffolds and the influence of cell behavior was evaluated.

## Figures and Tables

**Table 1:** Amino groups present in Ti disks.

Ti Disk	II	III	IV	V	VI
Free –NH_2_^a^	+	−	−	+	−

^a^ Measurements were carried out with a half-disk in duplicate. A positive sign is given if the absorbance is above 0.015 and a negative sign is given if the absorbance is below 0.007.

**Table 2:** X-ray photoelectron spectroscopy (XPS) elemental composition (at%) of Ti disks (VI) and (VII).

	Ti2p	O1s	Si2p	C1s	N1s	S2p
Ti disk VI	12.9	32.0	6.1	38.3	10.6	-
Ti disk VII	13.5	37.3	7.2	29.2	8.2	4.2

## Supplementary Materials

The following are available online at https://www.mdpi.com/2073-4360/12/4/770/s1, Figure S1: ^1^H NMR spectrum of 3,3’-diazidopivalic acid (2) in CDCl_3_, Figure S2: ^13^C NMR (SEFT) spectrum of 3,3’-diazidopivalic acid (2) in CDCl_3_, Figure S3: ^1^H NMR spectrum of benzyl-3,3’-diazidopivaloate (3) in CDCl_3_, Figure S4: ^13^C NMR (SEFT) spectrum of benzyl-3,3’-diazidopivaloate (3) in CDCl_3_, Figure S5: ^1^H NMR spectrum of benzyl-3,3´-diaminopivaloate (4) in D_2_O, Figure S6: ^13^C NMR (SEFT) spectrum of benzyl-3,3´-diaminopivaloate (4) in MeOD-*d*_4_, Figure S7: ^1^H NMR spectrum of benzyl-3,3´-bis(tert-butoxycarbonyl)aminopivaloate (5) in CDCl_3_, Figure S8: ^13^C NMR (SEFT) spectrum of benzyl-3,3´-bis(tert-butoxycarbonyl)aminopivaloate (5) in CDCl_3_. Figure S9: ^1^H NMR spectrum of 3,3´-bis(tert-butoxycarbonyl)aminopivalic acid (6) in MeOH-*d_4_*, Figure S10: ^13^C NMR (SEFT) spectrum of 3,3´-bis(tert-butoxycarbonyl)aminopivalic acid (6) in DMSO-*d*_6_, Figure S11: ^1^H NMR spectrum of 7 in CDCl_3_, Figure S12: ^13^C NMR (SEFT) spectrum of 7 in CDCl_3_, Figure S13: HSQC spectrum of 7 in CDCl_3_, Figure S14: ^1^H NMR spectrum of 1 in MeOD-*d*_4_, Figure S15: ^13^C NMR (SEFT) spectrum of 1 in MeOD-*d*_4_, Figure S16: HSQC spectrum of 1 in MeOD-*d*_4_, Table S1: NMR signal assignments of compound 7, and Table S2: NMR signal assignments of compound 1.
