# Supplementary material for: Dendritic Scaffold onto Titanium Implants. A Versatile Strategy Increasing Biocompatibility
Source: Polymers (Basel). 2020 Apr 1;12(4):770. doi: 10.3390/polym12040770 (PMC7240519; doi:10.3390/polym12040770)
Supplement: Supplementary file 1 [file polymers-12-00770-s001.pdf]

Dendritic Scaffold onto Titanium Implants. A Versatile Strategy Increasing Biocompatibility

Noemi Molina, Ana González, Donato Monopoli, Belinda Mentado, José Becerra, Leonor Santos-Ruiz, Ezequiel Perez-Inestrosa and Yolanda Vida

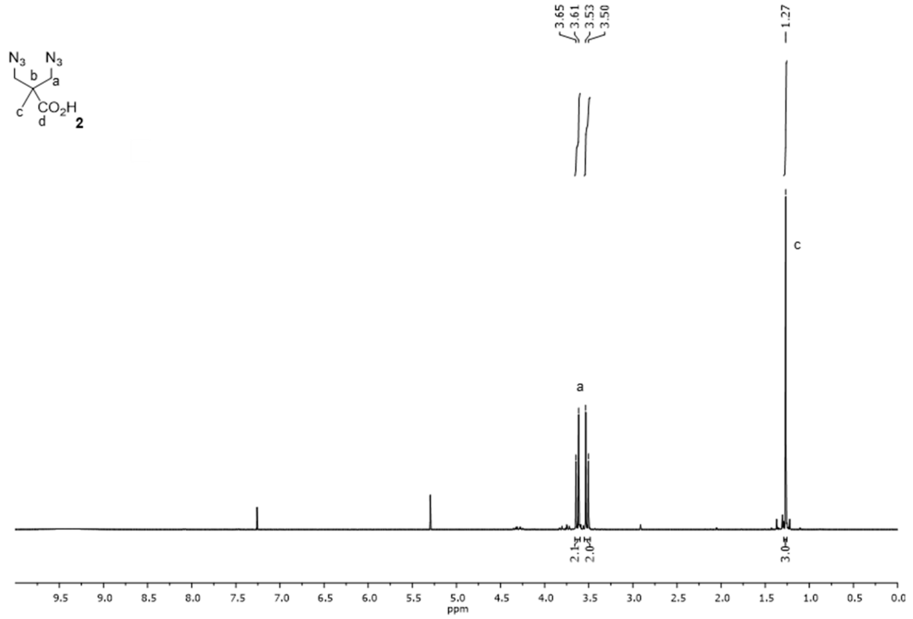

Figure S1. ^{1}H NMR spectrum of 3,3′-diazidopivalic acid (**2**) in $CDCl_3$.

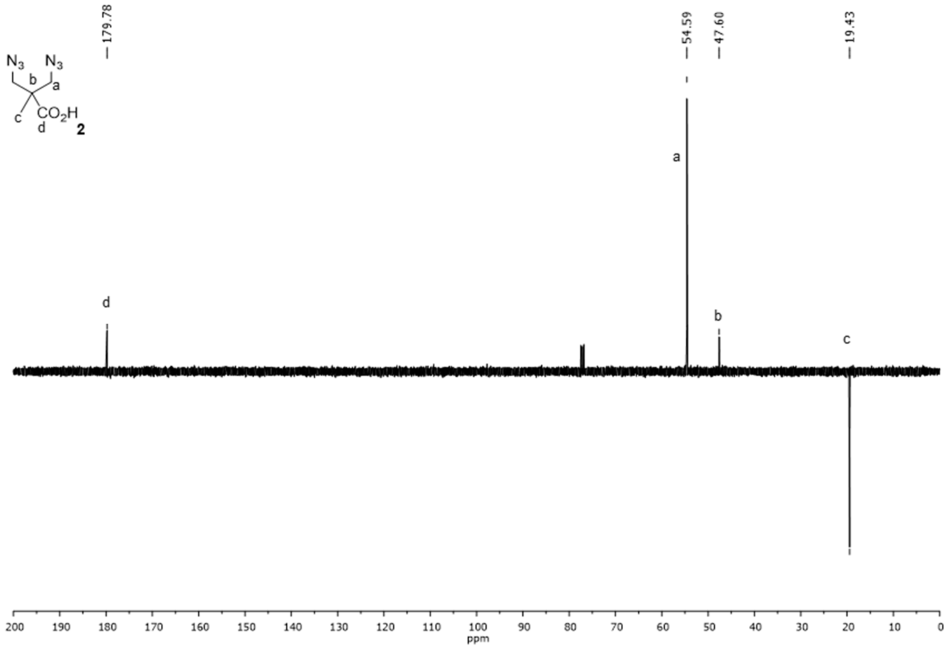

Figure S2. ^{13}C NMR (SEFT) spectrum of 3,3′-diazidopivalic acid (2) in $CDCl_3$.

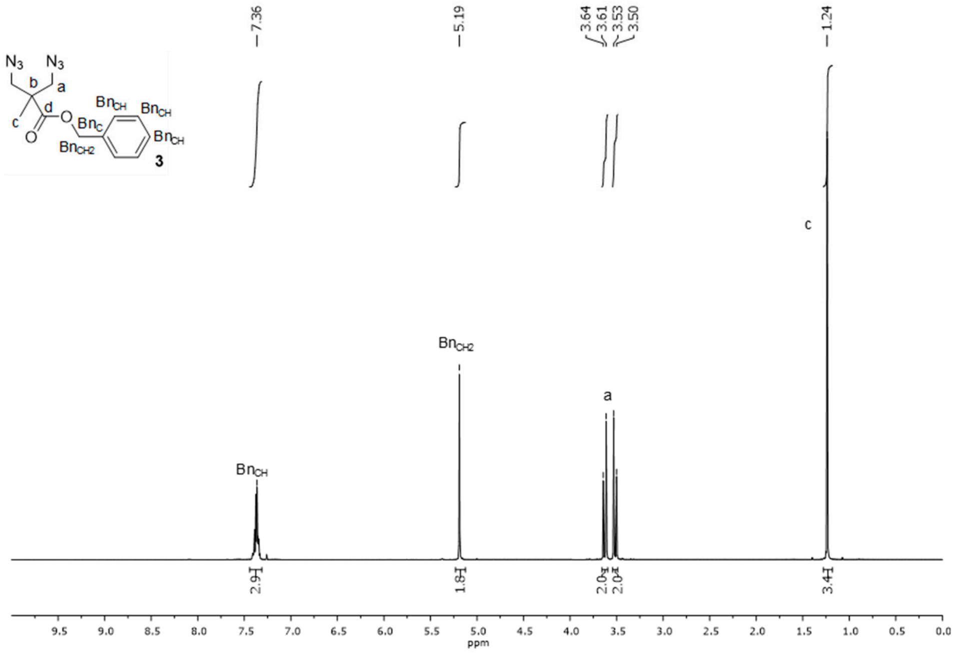

Figure S3. ^{1}H NMR spectrum of benzyl-3,3′-diazidopivaloate (**3**) in $CDCl_3$.

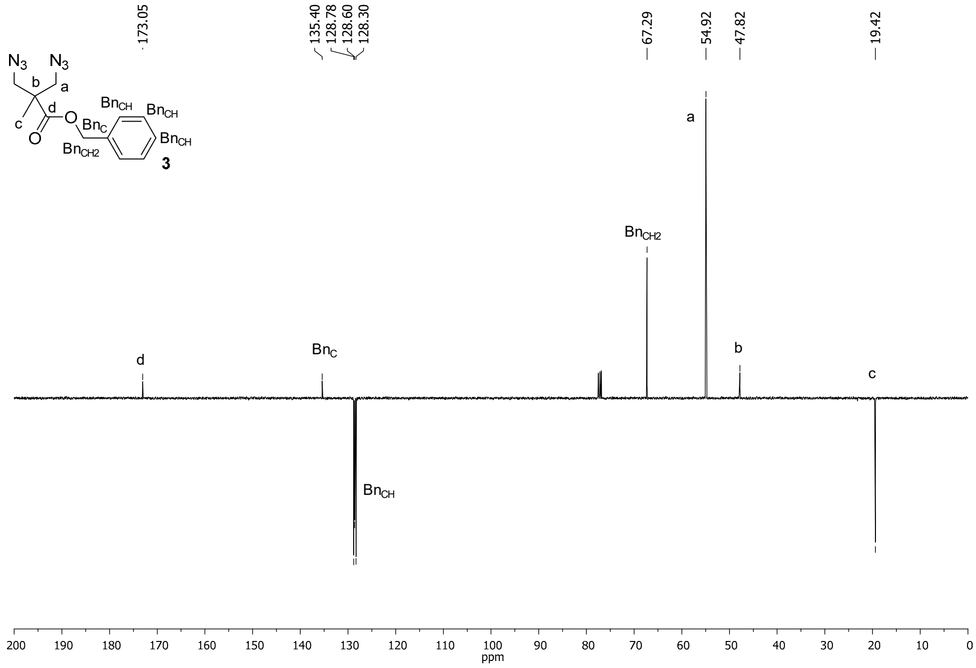

Figure S4. ^{13}C NMR (SEFT) spectrum of benzyl-3,3′-diazidopivaloate (**3**) in $CDCl_3$.

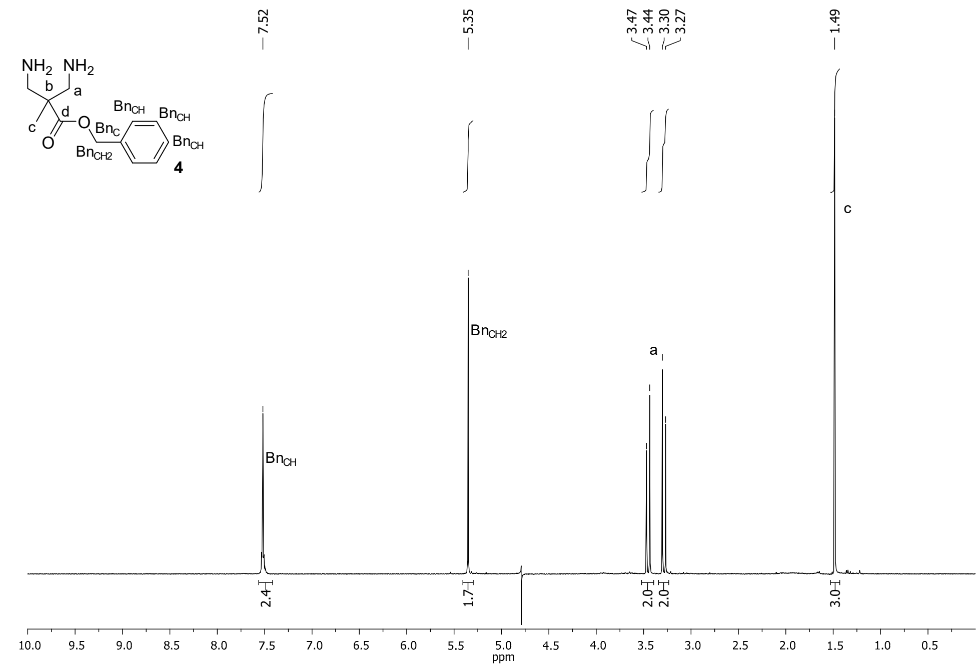

Figure S5. ^{1}H NMR spectrum of benzyl-3,3′-diaminopivaloate (**4**) in D_2O.

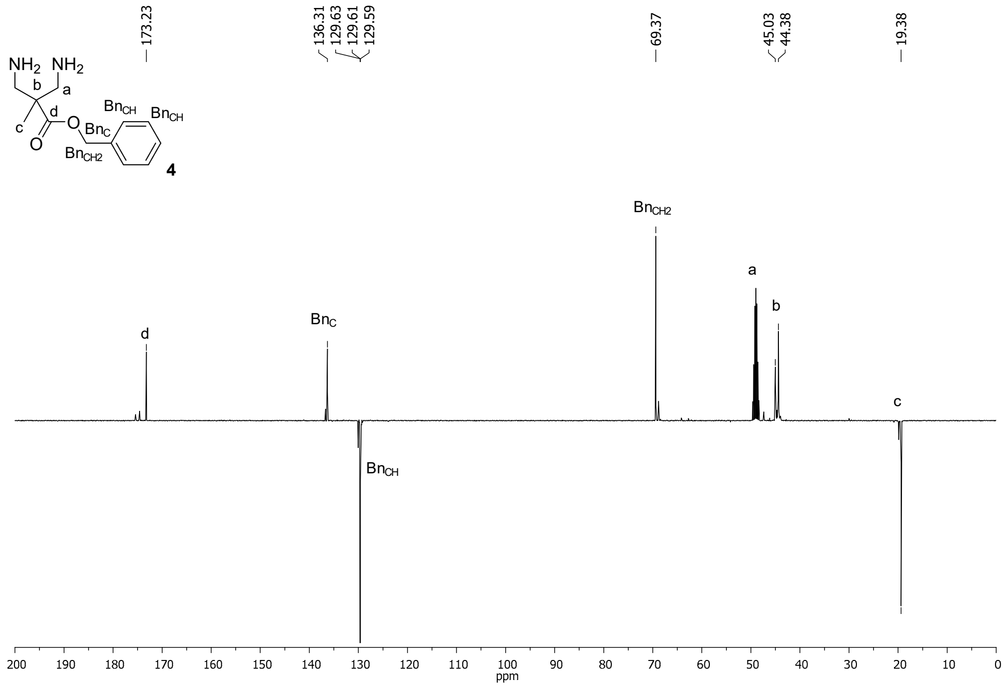

Figure S6. ^{13}C NMR (SEFT) spectrum of benzyl-3,3′-diaminopivaloate (**4**) in MeOD-d_4

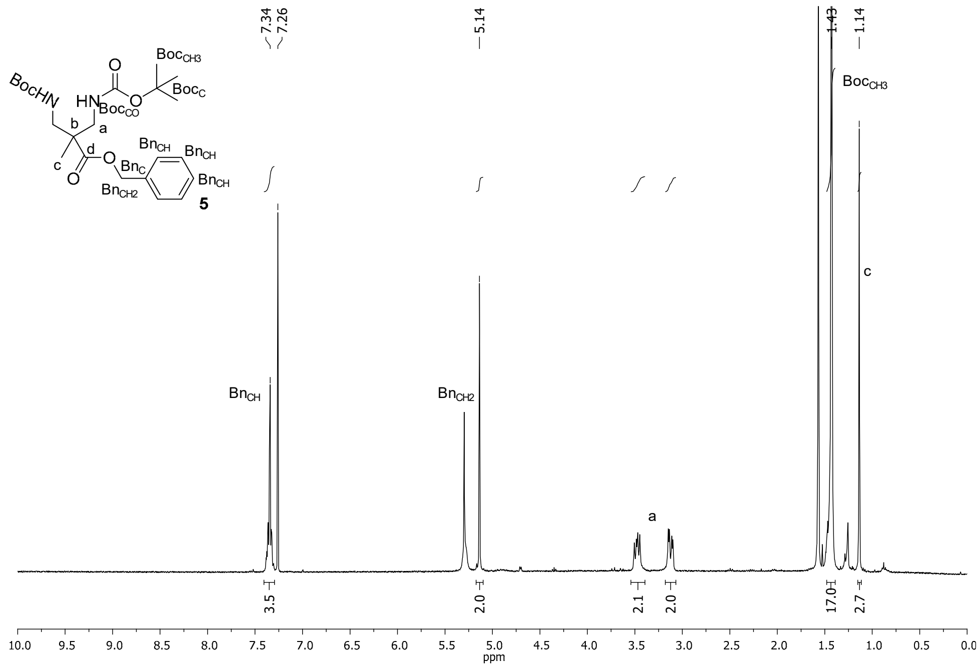

Figure S7. ^{1}H NMR spectrum of benzyl-3,3′-bis(tert-butoxycarbonyl)aminopivaloate (**5**) in $CDCl_3$.

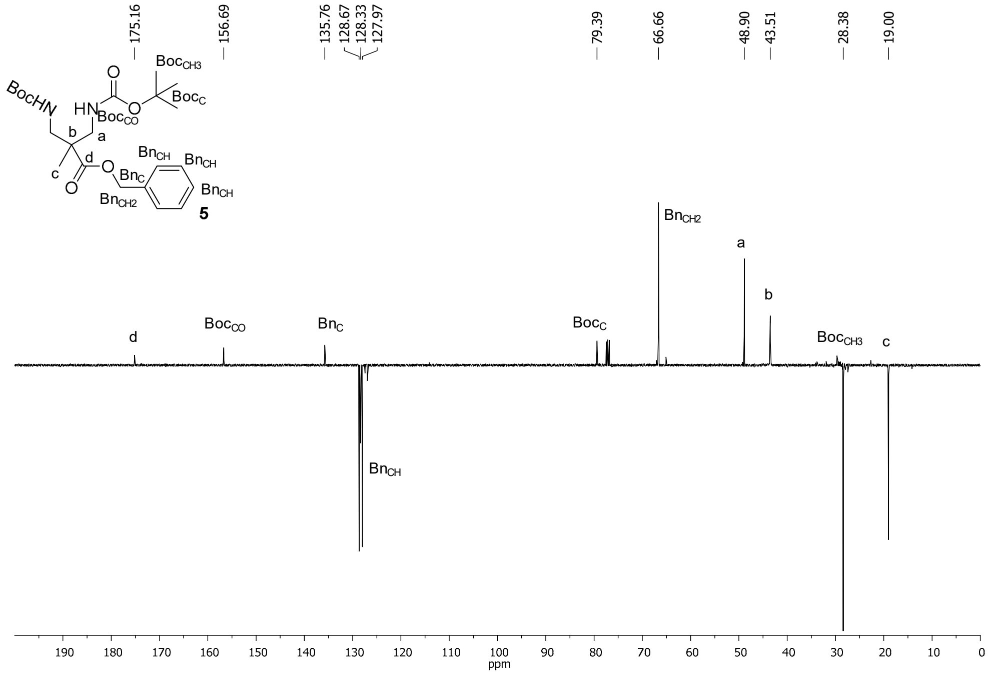

Figure S8. ^{13}C NMR (SEFT) spectrum of benzyl-3,3′-bis(tert-butoxycarbonyl)aminopivaloate (**5**) in $CDCl_3$.

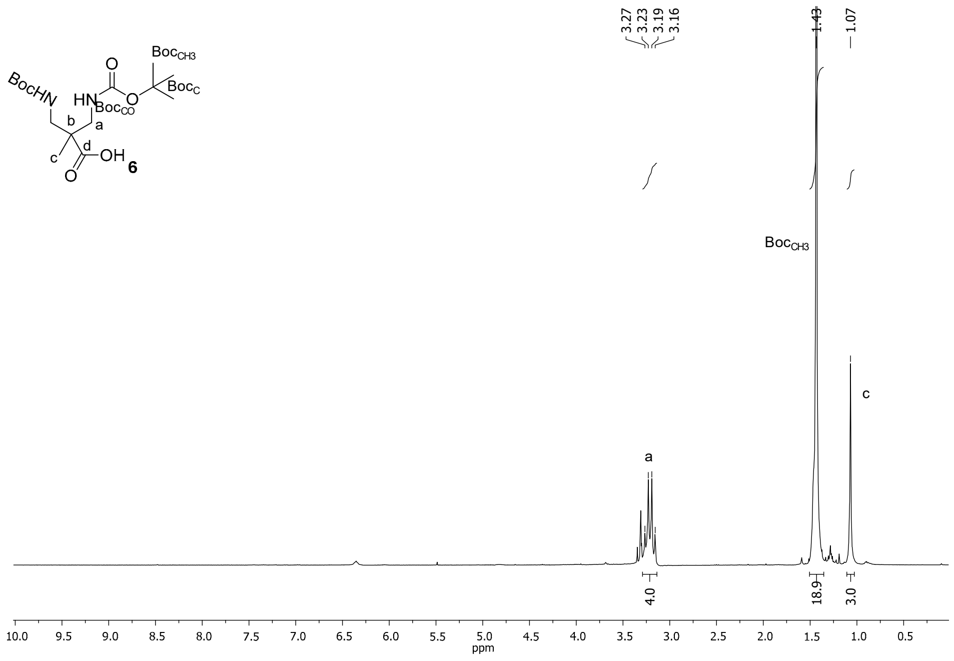

Figure S9. ^{1}H NMR spectrum of 3,3′-bis(tert-butoxycarbonyl)aminopivalic acid (**6**) in MeOH-d_4.

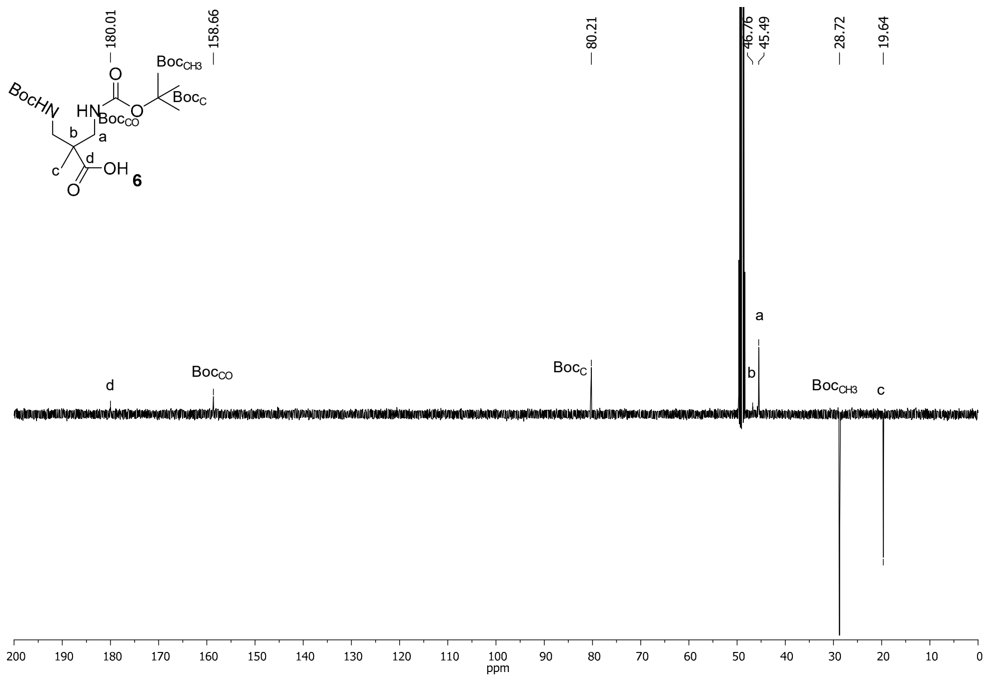

Figure S10. ^{13}C NMR (SEFT) spectrum of 3,3′-bis(tert-butoxycarbonyl)aminopivalic acid (**6**) in DMSO-d_6.

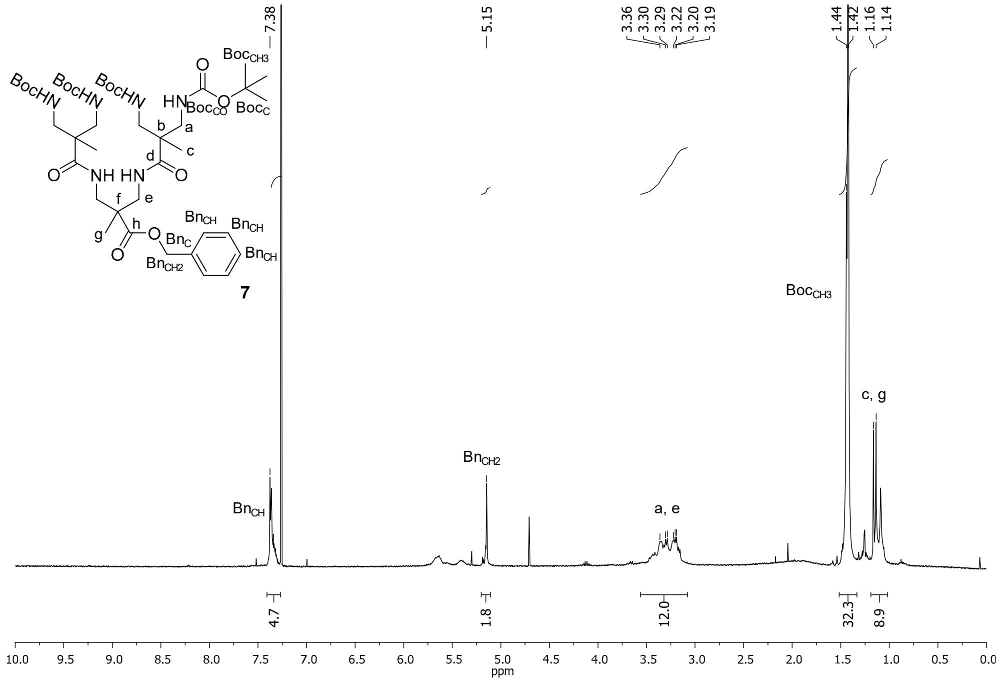

Figure S11. ^{1}H NMR spectrum of **7** in $CDCl_3$.

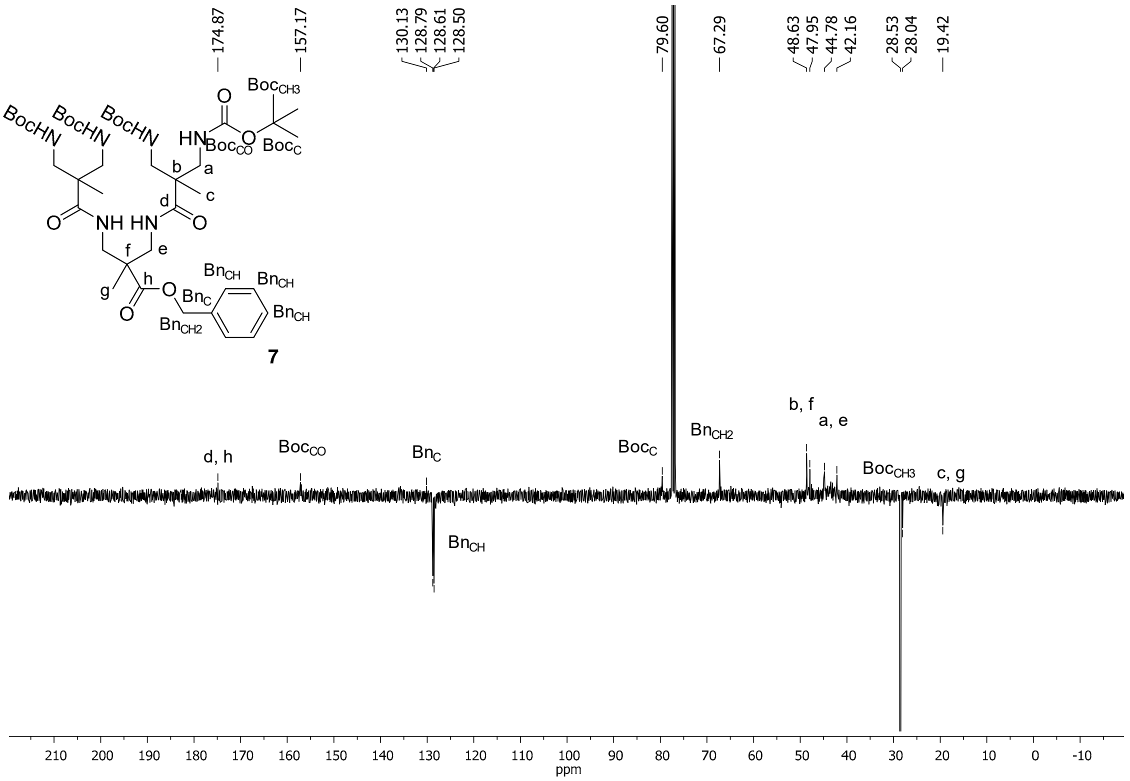

Figure S12. ^{13}C NMR (SEFT) spectrum of 7 in $CDCl_3$.

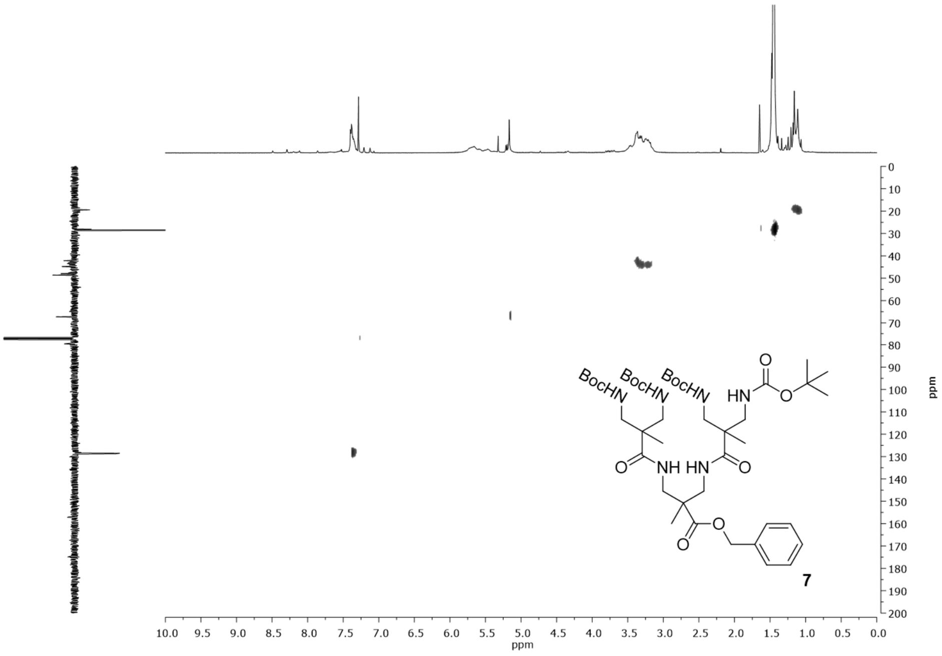

Figure S13. HSQC spectrum of 7 in $CDCl_3$.

Table S1. NMR signals assignments of compound **7.**

Position	^{1}H NMR (ppm)	^{13}C NMR (ppm)
**d,h**	-	174.9
**Boc$_{CO}$**	-	157.2
**Bn$_{C}$**	-	130.1
**Bn$_{CH}$**	7.40–7.29 (m, 5 H)	128.8, 128.6, 128.5
**Boc$_{C}$**	-	79.6
**Bn$_{CH2}$**	5.15 (s, 2 H)	67.3
**b,f**	-	48.6
**b,f**	-	48.0
**a,e**	3.51–3.06 (m, 12 H)	44.8
**a,e**	3.51–3.06 (m, 12 H)	42.2
**Boc$_{CH3}$**	1.44 (s, 36 H)	28.5
**c,g**	1.17–1.05 (m, 9 H)	19.4

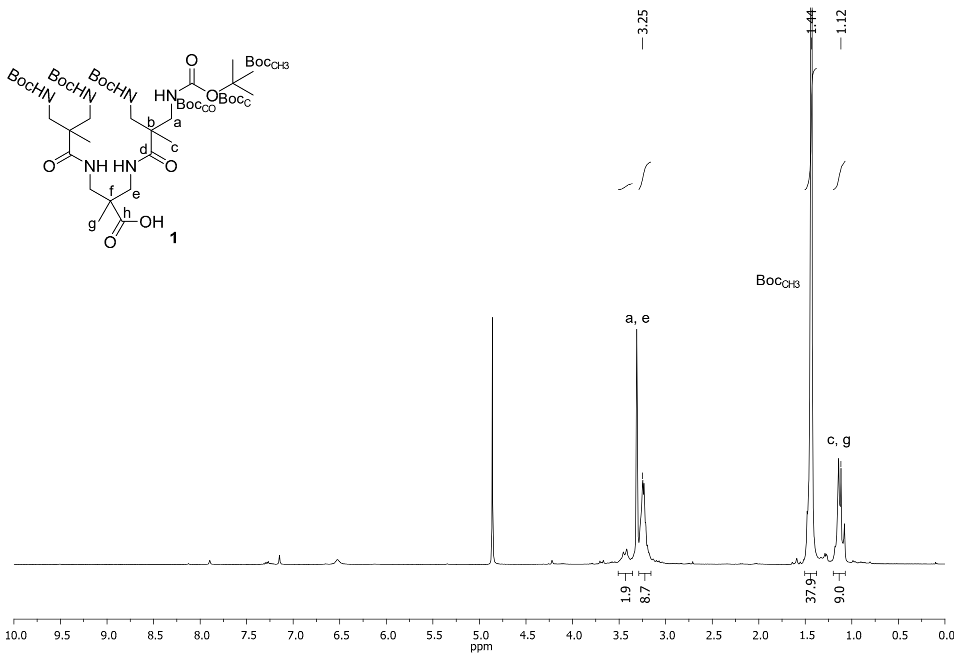

Figure S14. ^{1}H NMR spectrum of **1** in MeOD-*d*$_4$.

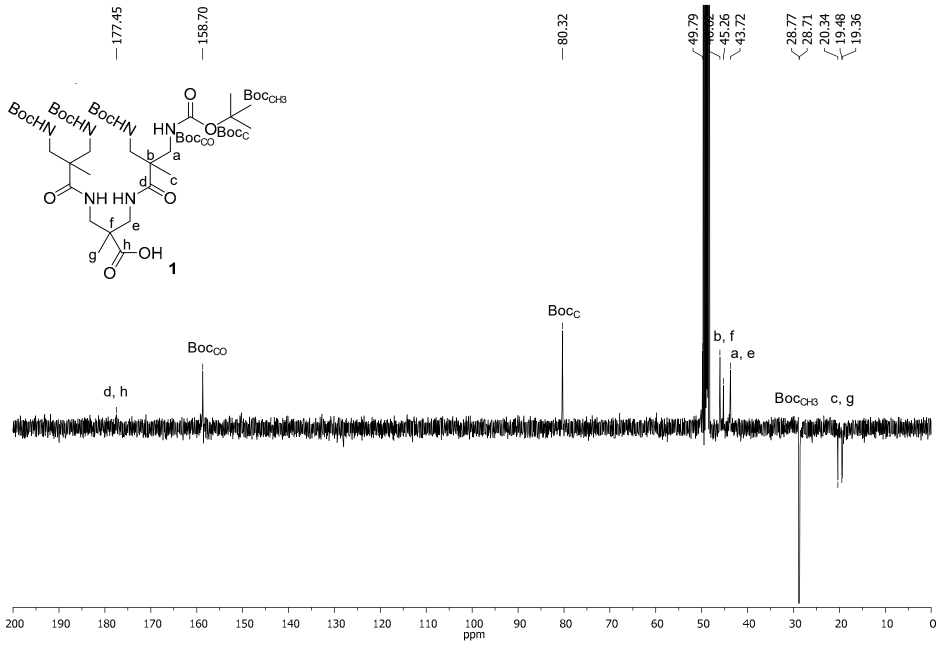

Figure S15. ^{13}C NMR (SEFT) spectrum of **1** in MeOD-*d*$_4$.

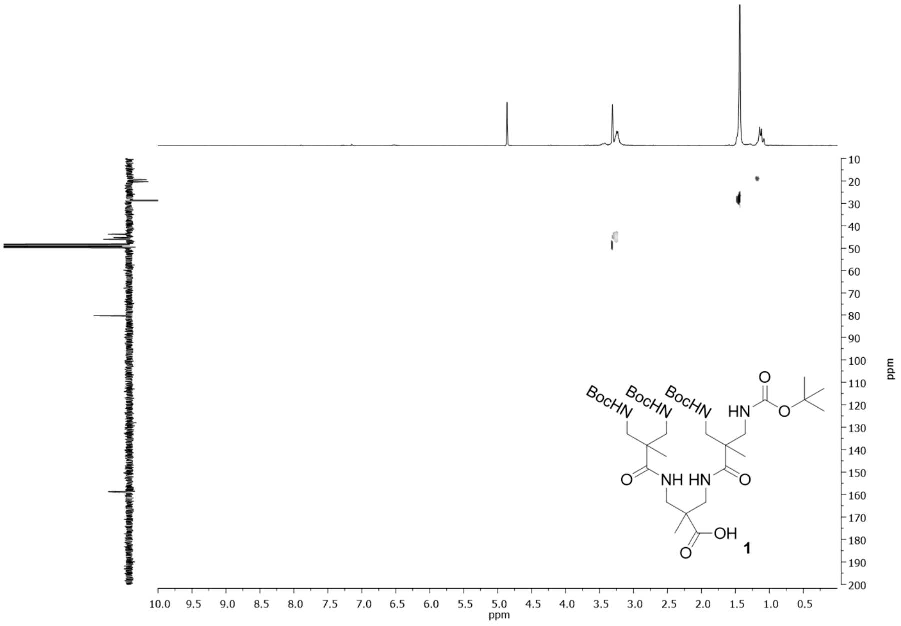

Figure S16. HSQC spectrum of **1** in MeOD-*d*4.

Table S2. NMR signals assignments of compound **1.**

Position	^{1}H NMR (ppm)	^{13}C NMR (ppm)
d,h	-	177.5
Boc$_{CO}$	-	158.7
Boc$_{C}$	-	80.3
b,f	-	49.8
b,f	-	46.0
a,e	3.48–3.20 (m, 12 H)	45.3
a,e	3.48–3.20 (m, 12 H)	43.7
Boc$_{CH3}$	1.44 (s, 36 H)	28.8
c,g	1.18–1.03 (m, 9 H)	20.3
c,g	1.18–1.03 (m, 9 H)	19.5
